# An innovative pharmacology curriculum for medical students: promoting higher order cognition, learner-centered coaching, and constructive feedback through a social pedagogy framework

**DOI:** 10.1186/s12909-021-02516-y

**Published:** 2021-02-05

**Authors:** Douglas McHugh, Andrew J. Yanik, Michael R. Mancini

**Affiliations:** Department of Medical Sciences, Frank H. Netter MD School of Medicine, Quinnipiac University, 370 Bassett Road, North Haven, CT 06473 USA

**Keywords:** Social pedagogy, Pharmacology, Conceptual framework, Medical students, Self-directed assessment, Feedback, Coaching, Constructivism, Networked learning, Communities of practice

## Abstract

**Background:**

Ongoing developments in medical education recognize the move to curricula that support self-regulated learning processes, skills of thinking, and the ability to adapt and navigate uncertain situations as much as the knowledge base of learners. Difficulties encountered in pursuing this reform, especially for pharmacology, include the tendency of beginner learners not to ask higher-order questions and the potential incongruency between creating authentic spaces for self-directed learning and providing external expert guidance. We tested the feasibility of developing, implementing, and sustaining an innovative model of social pedagogy as a strategy to address these challenges.

**Methods:**

Constructivism, communities of practice, and networked learning theory were selected as lenses for development of the model. Three hundred sixty-five first-year medical students participated between 2014 and 2018; they were introduced to pharmacodynamics and pharmacokinetics via 15 online modules that each included: learning objectives, a clinical vignette, teaching video, cumulative concept map, and small group wiki assignment. Five-person communities organized around the 15 wiki assignments were a key component where learners answered asynchronous, case-based questions that touched iteratively on Bloom’s cognitive taxonomy levels. The social pedagogy model’s wiki assignments were explored using abductive qualitative data analysis.

**Results:**

Qualitative analysis revealed that learners acquired and applied a conceptual framework for approaching pharmacology as a discipline, and demonstrated adaptive mastery by evaluating and interacting competently with unfamiliar drug information. Learners and faculty acquired habits of self-directed assessment seeking and learner-centered coaching, respectively; specifically, the model taught learners to look outward to peers, faculty, and external sources of information for credible and constructive feedback, and that this feedback could be trusted as a basis to direct performance improvement. 82–94% of learners rated the social pedagogy-based curriculum valuable.

**Conclusions:**

This social pedagogy model is agnostic with regard to pharmacology and type of health professional learner; therefore, we anticipate its benefits to be transferable to other disciplines.

**Supplementary Information:**

The online version contains supplementary material available at 10.1186/s12909-021-02516-y.

## Background

Increasing attention is being paid to learner-centered coaching and helpful feedback as educational strategies relevant to various health education contexts [1–4]. This is consistent with the larger trending reform movement in health professions education that emphasizes self-regulation coupled to the development and practice of adaptive mastery [5, 6]. Lefroy et al. defined helpful feedback as, “a supportive conversation that clarifies the trainee’s awareness of their developing competencies, enhances their self-efficacy for making progress, challenges them to set objectives for improvement, and facilitates their development of strategies to enable that improvement to occur” [7]. Such feedback supports self-regulation by responding to learners’ proactive seeking of formative commentary on the outcomes of their actions from sources external to themselves. Here we contribute a theory-informed model of social pedagogy to this movement. It establishes a promising means not only for guided discovery and engagement of learners with complex basic science content, but also to teach students to think critically and use available resources for their own learning. Of note, while this learning model is agnostic with regard to basic science content and type of health professional learner, it was developed and implemented in the context of teaching first-year medical students the general principles of pharmacology.

### Challenges in current approaches for teaching and learning pharmacology

Pharmacology is an intimidating subject in medical education curricula [8]. Teaching it involves articulating to learners meaningful connections between molecular and cell biology, biochemistry, physiology, and clinical medicine. Many medical schools expect students to learn the underlying general principles of pharmacokinetics (the effects of the body on drugs) and pharmacodynamics (the effects of drugs on the body) in a relatively short period [9]. In addition, the amount of therapeutically relevant information available to teach becomes greater each year [10]. Students report anxiety and difficulty assimilating the extensive pharmacological knowledge required of them [9, 10]. Many pharmacology curricula feature a lecture-heavy format or a problem-based learning (PBL) approach.^1^ The latter requires, essentially, students to teach themselves the necessary knowledge and its application using clinical cases and exploration of self-identified learning topics. Both teaching modalities have challenges (Table 1) and fall short of their fullest potential [9, 11–15].

**Table 1:** Challenges with Lecture- and PBL-based approaches

Lecture-based approaches	PBL-based approaches
• Substantial volume of material • Information transmission-oriented • Synchronous in-person or recorded • Faculty-centric • Passive • Memorization heavy • Limited peer teaching, peer interactions, peer co-creation of knowledge	• Self-directed learning permits students to gloss over difficulty topics • Limited feedback from faculty facilitators • Insufficient familiarity with subject matter to self-identify knowledge gaps • Limited faculty guidance to foster cognitive bootstrapping, scaffolding, and flexibility • Absence of a conceptual framework for approaching the discipline

Challenges in current formats used in pharmacology education

Traditionally, pharmacology is taught as a lecture-based, stand-alone pre-clerkship course. Students find it to be overwhelming and feel pressured to memorize everything presented to them [8, 16]; skewing learning towards *remembering* and *understanding* in Bloom’s cognitive taxonomy. Memorization does not translate information into knowledge or facilitate its application in clinical contexts and students often do not completely comprehend the integration and operational basis of pharmacological therapeutics. To address the short-comings of passive, information transmission-oriented approaches medical schools have moved to active-learning such as PBL [17]. However, PBL has limitations for teaching and learning complex basic science content with clinical applications such as pharmacology, including learners failing to master basic science concepts in clinically oriented, case-based learning [8, 18]; limited feedback from faculty, due to self-directed learning as an essential attribute of PBL [18]; and students glossing over difficult pharmacology concepts in favor of easier topics [8]. These challenges are exacerbated when faculty facilitators are uncomfortable discussing clinical cases from a pharmacokinetic and pharmacodynamic standpoint [19].

First- (M1) and second-year (M2) medical students also may not be sufficiently familiar with core pharmacology concepts to let them pose pertinent questions to understand the actions of drugs from a molecular perspective to the level of the human patient, and they may be unaware of these knowledge gaps in the absence of guidance from expert faculty [20]. Both transmission-inclined approaches and PBL limit students’ opportunities to develop ‘cognitive flexibility’ – the ability to internalize information that will allow them to independently solve future variations of pharmacology problems addressed during teaching sessions [8].

### Social pedagogy as a promising alternative for learner-centered coaching and helpful feedback

The diffusion of internet connectivity, mobile devices, and participatory media has contributed to peer-based and networked forms of learning. Social pedagogy emerges functionally from this and emphasizes guided discovery, collegial co-creation of knowledge, and learners leading each other [21, 22]. This new approach calls attention to the relationships and interactions within which learning takes place. Learning is peer-based and co-created, rather than hierarchical and expert-dependent. Compared to PBL, social pedagogy offers more support and scaffolding for learning [23]. It offers an attractive alternative in a world with abundant knowledge and learning needs, and the availability of media that facilitate learning and engagement. Daniel Pratt’s work on conceptions of teaching provides a succinct synopsis of existing frameworks and perspectives that are useful in contextualizing social pedagogy; in particular, Pratt’s comments on *transmission* (“the learner is a ‘container’ that is to be filled with knowledge”) and *developmental* (“the learner develops increasingly complex and sophisticated ways of reasoning and problem-solving”) [24, 25].

Our objective was to study whether social pedagogy has the potential to address challenges inherent to lecture- or PBL-based learning formats by allowing medical students to understand and apply the concepts of pharmacology. We tested the feasibility of developing, implementing, and sustaining a model of social pedagogy, within which pharmacology learning was nested, as a strategy for teaching M1 medical students at the Frank H. Netter MD School of Medicine (FHN SOM) at Quinnipiac University. The expected outcomes were for students to (1) acquire a conceptual framework for approaching pharmacology as a discipline, and (2) to repeatedly self-assess their developing mastery of its underlying principles across Bloom’s cognitive taxonomy with case-based wiki exercises. Bloom’s cognitive taxonomy, since its introduction in 1956 and after its revision in 2001, is usually visualized as a hierarchical ordering of cognitive skills related to teaching and learning. A few of criticisms of this include that the bottom-to-top order conveys an impression of ranked value, and that these cognitive processes are discrete and separable [26, 27]. That aside, as a classification system well-known among educators, we used it to direct attention to what students can do at the various stages of their learning processes.

## Methods

### Research team

The research team comprised three members with firsthand experience of the social pedagogy model. Two were M2 medical students (AY and MM) and one was a pharmacology faculty member with involvement in the model’s design and experience in qualitative methodology and assessment in medical education (DM).

### Theory orientation

We selected *constructivism*, *networked learning theory* and *communities of practice theory* as lenses that informed the development and evaluation of our social pedagogy model for pharmacology learning (Table 2). *Constructivism* holds that, through personal experience and reflection, humans learn by forming their own knowledge and understanding; information is actively constructed rather than passively absorbed [28]. Instructional scaffolding of Bloom’s higher-level cognitive processes from lower-level ones is an application of constructivism [23, 25]. An essential element of *networked learning* theory is that much of the learning happens in online peer networks [29], with educators guiding learners to information sources and answering select questions, on an as-needed basis, to foster co-construction and sharing of knowledge. Learners are encouraged to seek out information on their own online and report what they find. This contributes to a ‘community of practice’ (CoP) around the growing shared cognition. CoPs are formed by individuals with an authentic connection to one another and who collaborate for the sake of mutual improvement through recurrent, planned interactions. Belonging to a CoP implies members’ commit to participate in joint activities, share information and help one another, and develop an accessible cache of resources (experiences, materials, instruments… etc.) to address common challenges [30].

**Table 2:** Education theories informed educational methods

	Constructivism	Communities of Practice	Networked Learning
**Key Features**	• Learning is an active, contextualized process of constructing knowledge through experience. • Active creators of own knowledge. • Ask questions, explore, assess what we know. • Experience and reflection.	• Learning is a social, collaborative process. • People who have common interests collaborate over an extended period of time. • Contribute ideas and strategies, determine solutions. • Embrace share responsibility.	• Learning is perceiving connections between fields, ideas, and concepts. • Use of internet technologies to learn and share information. • Asynchronous, online, peer networks. • Faculty guide discovery and answer key questions as needed. • Learners encouraged to seek and share information on their own.
**Educational Methods**	• Individual learners answer 1 of 5 case-based, wiki assignment questions across Bloom’s taxonomy. • Reflection upon other learners’ posts. • Individual learners post 1 peer-teaching, follow-up comment.	• Small group wiki communities of *n* = 5 learners. • Collaboration extended over M1 academic year. • Social learning; Shared responsibility.	• Blackboard® Wiki Tool. • 15 *formative* small group wiki assignments; 1 *summative* individual wiki assignment. • Learners contribute ideas, strategies, solutions, comments, and ask additional questions of one another.

Elements of constructivism, communities of practice theory, and networked learning theory used in the design of the social pedagogy model for pharmacology learning

### Social pedagogy-based pharmacology curriculum

At FHN SOM, M1 students are introduced to pharmacodynamics and pharmacokinetics in the context of over-the-counter medications and human organ systems. This is achieved through 15 online modules (Additional file 1) and integrated pharmacology lectures. The modules include learning objectives, a clinical vignette, teaching video, cumulative concept map, and small group wiki assignment (Additional file 2). The integrated pharmacology lectures, through blended learning [31], reinforce concepts taught in the modules and move learners from a simple to more complex mastery of pharmacokinetics and pharmacodynamics as they relate to specific organ systems.

### Social pedagogy-based pharmacology wiki communities

Small groups organized around wikis are at the center of the social pedagogy curricular model. Wikis are websites that permit collaborative modification, extension, or deletion without a defined owner or leader, allowing content to emerge according to the users’ activities. M1 students are assigned randomly to small group communities of 5 learners using the *random enroll* feature of the Blackboard® learning management system (Blackboard, Inc.; Washington D.C.). Each group is provided with their own copy of a wiki assignment using the Campus Pack Wiki tool (Learning Objects, Inc.; Washington D.C.). Permissions are set such that only the 5 learners belonging to a given wiki group can view or edit the group’s assignments. Faculty can access assignments and information across all wiki groups. Group members have approximately 1 week per wiki assignment to answer asynchronously 1 of 5 (sometimes multi-part) case-based questions and provide at least 1 peer-teaching follow-up comment in response to other students’ posts.

**Fig. 1:**
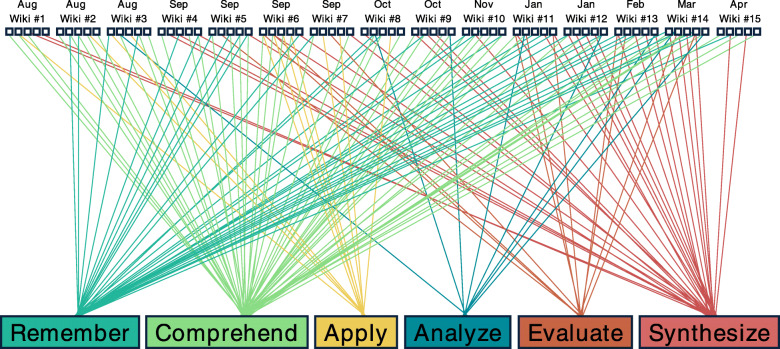
Mapping of wiki assignment questions to Bloom’s cognitive taxonomy. Mapping of questions 1–5 from wiki assignments #1–15 to the six tiers of Bloom’s cognitive taxonomy; each small square (☐) represents an individual question

Wiki small groups self-determine which member will answer a given question. Each of the 15 wiki assignments over the length of the curriculum were designed to touch on several of Bloom’s cognitive taxonomy levels, to ensure learners were repeatedly challenged to engage in lower-to-higher order thinking (Fig. 1). Blackboard® provided the technological means for faculty to monitor wiki activity and weave in feedback and coaching during on-going learning. Wiki assignments were released on Fridays, answers to the 5 questions were due by the following Tuesday, and peer-teaching follow-up comments were due on Thursday of the same week.

### Learner assessment

We developed an assessment system for the social pedagogy model of pharmacology learning:

- 15 *formative* small group wiki assessments (one per module; August to April) with the goal of monitoring student learning to provide ongoing feedback that could be used by students to regulate their learning and help faculty recognize where students were struggling and provide coaching promptly.
- 1 *summative* individual, final wiki assessment (one per student; May) with the goal of providing an opportunity for learners to demonstrate they had acquired a conceptual framework for approaching the discipline such that they could evaluate and interact competently with drug information not featured elsewhere in the curriculum.
- 11–17 *summative* faculty-developed single-best-answer multiple-choice questions (MCQs) with the goal of providing internal, objective metrics that complemented the wiki assessments. The assessment culture at FHN SOM is such that MCQs are expected as an efficient way of reassuring key stakeholders (i.e., learners and faculty) that students are being prepared adequately for future practice and United States medical licensing exams. Questions tested learner competence with pharmacokinetic and pharmacodynamic principles across six end-of-block summative exams (occurring after 6 weeks of foundational science teaching, typically) between 2014 and 2018. MCQ items adhered to best practices for item writing and were reviewed by 4 pharmacology faculty members to ensure consistency with learning objectives [32].

Four FHN SOM faculty with PhDs in pharmacology set the standard for the pass/fail rubric for the wiki exercises (Additional file 3). This involved developing definitions of minimally competent responses, and review and acceptance of faculty-developed example answers to each wiki assignment [33]. Faculty members made *formative* pass/fail judgments using the rubric relative to each small group wiki assignment and the agreed-upon faculty-developed example answers.

For the final, summative wiki assignment, individual students chose 1 of 10 over-the-counter (OTC) drug/supplements that had not previously been featured in the curriculum (Additional file 4). Students had to evaluate one claimed therapeutic benefit using their knowledge of pharmacokinetics and pharmacodynamics and the consumer version of the drug/supplement facts provided at point-of-sale. Learners were given 1 week to complete the assignment and permitted an unlimited number of revisions during that time. The four pharmacology faculty members made *summative* pass/fail judgments using the rubric and faculty-developed example answers for the 10 OTC drugs/supplements.

### Participants and setting

Between 2014 and 2018, M1 students from FHN SOM were assigned randomly to small group wiki communities and participated in the social pedagogy-based pharmacology curriculum as a requirement for the ‘Foundations of Medicine I’ course. Participants totalled 365 (90 each 2014–2015; 2015–2016; 2016–2017; and 95 in 2017–2018) and responded to the evaluation question, “Please rate the value of the pharmacology module/wiki learning activity and resources in helping you achieve the learning objectives” on a 4-point scale.

### Qualitative analysis of wiki assignments

The social pedagogy model’s wiki assignments were explored using abductive, progressive focusing, which combines inductive and deductive approaches [34]. Progressive focusing permits qualitative findings to emerge from analysis of empirical phenomena and its interaction with theory [35]. Wiki assignment data sets were reviewed independently and coded provisionally by AY, MM, and DM. Initial codes were derived from the preexisting frameworks of constructivism, networked learning theory, and communities of practice theory [28–30]. We analyzed the content of the wiki assignments to deduce patterns arising from the presence and absence of these initial codes. Further codes were constructed to capture unanticipated observations, processes, and behaviors. These were derived inductively by constant comparison [36]. This approach allowed the main aspects of our social pedagogy model to be integral to the process while permitting data-derived themes to surface. After the first 36 wiki assignments were coded, the research team met to discuss the coding schemes and conceptual memos. The researchers met several more times and refined codes using an iterative approach to cluster categories and identify pertinent concepts, which were further refined into major themes. NVivo 12 qualitative analysis software (QSR International Inc., Burlington MA, USA) was used to facilitate these coding processes. Mean percentage prevalence (+/− standard deviation) of learner responses, that aligned with the thematic behaviors discerned during qualitative analysis, were quantified in the 73 wiki small groups that interacted with formative wiki assignments 1–15 and the final, summative wiki assignment between 2014 and 2018.

## Results

### Inter-rater reliability

We calculated Fleiss’ kappa coefficient for inter-rater reliability [37] of the four faculty pharmacologists using a random sample of 40 wiki assignments. This showed a kappa score of 0.97.

### Summative MCQs

Standard setting for ‘Foundations of Medicine I’ summative exams is the responsibility of the FOM grading committee, which is composed of 17 faculty members (basic or clinical science). Standard setting included consideration of the consequences to learners, defining a minimally competent response, MCQ item statistical analysis (Additional file 5), and the z-score distribution of learner performance. Between 2014 and 2018, all M1 students were judged to be competent with regard to the pharmacology summative MCQs.

### Learner acceptance

Between 2014 and 2018, a range of 82–94% of learners in each cohort rated the social pedagogy-based pharmacology curriculum either extremely or somewhat valuable in helping them meet associated learning objectives (Table 3).

**Table 3:** Learner evaluation of pharmacology module/wiki learning

4-Point Scale Evaluation Question	M1 students 2014–2015 (*n* = 90)	M1 students 2015–2016 (*n* = 90)	M1 students 2016–2017 (*n* = 90)	M1 students 2017–2018 (*n* = 95)
Extremely Valuable	53.1%	41.9%	51.6%	49.4%
Somewhat Valuable	40.6%	41.8%	30.6%	35.9%
Neutral	6.3%	11.6%	12.5%	10.5%
Not Valuable	0%	4.7%	5.3%	4.2%

Percentage response on a 4-point scale to the evaluation question, “*Please rate the value of the pharmacology module/wiki learning activity and resources in helping you achieve the learning objectives”* for M1 students 2014–2018

### Formative and summative assessment of wiki assignments

The percentage of students judged not competent in formative small group wiki assignments ranged from 7.8 to 15.6%. The predominant reason for not passing was learners being late with their answers (Additional file 6). All learners demonstrated competence in the final, summative individual wiki assignments. One learner in 2015 failed initially, due to lack of timely submission.

### Qualitative findings

We explored free text comments from the wikis to assess the extent to which leaners acquired and applied a conceptual framework for approaching pharmacology as a discipline, and the extent to which they self-assessed their developing mastery of its underlying principles using Bloom’s taxonomy with case-based wiki exercises. Illustrative quotes for selected concepts are shown; learner names have been replaced with pseudonyms.

#### Learners acquired a conceptual framework for approaching pharmacology as a discipline

We functionally defined a conceptual framework as, “a network of interlinked concepts where intersecting, related abstractions provide an interpretive context that facilitates comprehension, analysis, and explanation of observed phenomena.” [38–41]. Table 4 displays coding of one representative final wiki assessment submission, broken up using ellipses ‘…’; an uninterrupted version of this individual student response can be viewed in Additional file 4. In it, the learner crisscrossed all six levels of Bloom’s taxonomy and employed an array of conceptual framework elements to interpret, evaluate, and synthesize conclusions about the stated pharmacological benefits of the OTC medication, Dream Water. As noted earlier, all learners demonstrated individual competence with this summative outcome.

**Table 4:** Coding of a representative example of a summative final wiki assessment

**Bloom’s Cognitive Taxonomy**	**Conceptual Framework Elements**	**Pharmacodynamics Excerpts from a representative Summative Wiki**
***Remember*** - identify, explain, define, recall, recognize, describe	**Distribution**	“GABA (ɣ-aminobutyric acid) is a neurotransmitter widely distributed within the central nervous system, and represents the major element in neuronal inhibition (Julio-Pieper et al. 2013)...”
***Comprehend*** - relate, categorize, explain		
***Evaluate*** - searching, supporting, convincing	**Drug-Receptor Interactions**	
***Synthesize*** - composing, wiki-building		
***Remember*** - identify, explain, define, recall, recognize, describe	**Drug-Receptor Interactions**	“…There are two main GABA receptors within the brain: GABA_A_ and GABA_B_. GABA_A_ is the most well-studied of the two. It is a ligand-gated chloride ion channel expressed on the post-synaptic side of the cleft, with orthosteric and allosteric binding sites. With GABA release from the pre-synaptic terminal, and binding to GABA_A_, the Cl^−^ channel is activated and Cl^−^ enters the neuron, effectively hyperpolarizing the membrane and increasing the threshold for action potential generation…”
***Evaluate*** - assess, consider	**Drug-Response Relations**	
***Synthesize*** - composing, wiki-building		
***Comprehend*** - relate, associate, contrast	**Drug-Receptor Interactions**	“…Unlike GABA_A_, GABA_B_ is a G-protein-coupled receptor. GABA signaling here decreases calcium conductance, inhibits cAMP production, increases K^+^ efflux – the net effect of which will hyperpolarize and reduce the frequency of post-synaptic action potentials (Kumar et al. 2015. *Neuropharmacology*. 97:414–425)…”
***Evaluate*** - searching, supporting, convincing	**Drug-Response Relations**	
***Synthesize*** - composing, wiki-building		
***Analyze*** - appraising	**Drug-Response Relations**	“…by triggering the inhibitory effects of GABAergic neurons in the central nervous system, the GABA added to Dream Water could potentially exert anxiolytic and myorelaxant effects (Richard and Foster 2003; Chou et al. 2008; Mazzoli et al. 2010)…”
***Evaluate*** - hypothesizing		
***Synthesize*** - composing, wiki-building		
**Bloom’s Cognitive Taxonomy**	**Conceptual Framework Elements**	**Pharmacokinetics Excerpts from a representative Summative Wiki**
***Remember*** - explain, recall, describe	**Elimination – Metabolism & Excretion**	“…After exerting its function within the synaptic cleft, GABA experiences reuptake, whereby the presynaptic terminal and surrounding supporting glia cells reabsorb it in an exchange with sodium. The GABA which is reabsorbed by the neuron can be reused, while the GABA reabsorbed by the glia is metabolized to succinic semialdehyde and must be regenerated by GAD. (Cherlyn et al. 2010; Femenía et al. 2012)…”
***Comprehend*** - relate, associate, contrast		
***Evaluate*** - searching, supporting, convincing		
***Synthesize*** - composing, wiki-building		
***Comprehend*** - relate, associate, contrast	**Absorption**	“…Exogenously administered GABA, as in the case of Dream Water, would be subject to the challenges of absorption, distribution, metabolism, and excretion, as any exogenous substance is. GABA’s capacity to traverse the blood-brain barrier (BBB) is controversial (Boonstra et al. 2015). The bulk of evidence supports the impermeability of BBB to GABA, although Boonstra et al. (2015) report that “BBB permeability to GABA could diminish with increasing age.” In addition, mouse model studies have demonstrated evidence of GABA-facilitated transport across the BBB; however, measurements of GABA efflux were 17x greater than GABA-facilitated influx (Kakee et al. 2001). The presence of equivalent GABA transporters in the human blood-brain barrier has yet to be established. Moreover, Knych et al. (2015) demonstrated in a horse animal model that exogenous oral GABA – with a half-life of 25 min, bioavailability of 9.8%, and urine concentration peaking at 3 h – had no discernable behavior effect. This means that half of the given GABA had been metabolized within 25 min, and that of all of the GABA administered, only 9.8% was available as active within the body. Given that no behavioral changes where seen with exogenous oral GABA, but was with IV, it appears that it did not appreciably enter the cerebrospinal fluid to exert its effects…”
***Apply*** - applying, judging	**Bioavailability**	
***Evaluate*** - searching, supporting, convincing	**Distribution**	
***Synthesize*** - composing, wiki-building	**Elimination – Metabolism & Excretion**	
	**Elimination – Clearance & Half-life**	
***Apply*** - judging, determining	**Drugs and the Blood-Brain Barrier**	“…Therefore, the ultimate benefit of the GABA added to Dream Water may be in question. A Phase I clinical trial to study exogenous GABA in humans is currently recruiting participants (https://clinicaltrials.gov/ct2/show/NCT01917760)…”
***Evaluate*** - assessing, concluding	**Peripheral Restriction of Drugs**	
***Synthesize*** - composing, wiki-building		

Coding of a single, representative example of a summative final wiki assessment with respect to Bloom’s cognitive taxonomy and pharmacological conceptual framework elements

We also explored the degree to which our approach reflected key principles of social pedagogy, and found that learners were (1) routinely seeking assessment and sharing information with one another; (2) identifying peer misperceptions or errors and providing gentle correction and guidance; (3) reframing what they have learned at the molecular or cellular level in a whole patient context; (4) transferring learning to their weekly primary care clinical medical student home (MeSH) sites and applying it with patients where appropriate and under supervision; (5) providing evidence-based answers by accessing, screening, analyzing, and citing the literature base; and (6) asking their own self-generated questions to one another. Additional characteristics of social pedagogy demonstrated during wiki interactions included faculty providing external guidance to foster cognitive bootstrapping, scaffolding, and flexibility in learners; and unobtrusively monitoring learner progress and interactions online. We quantified the mean prevalence of these behaviors among the 73 wiki small groups (Table 5). Collectively, these attributes that emerged support our approach as an authentic and feasible application of social pedagogy in the education of medical students in their pre-clinical years.

**Table 5:** Percentage prevalence of learner responses that aligned with thematic behaviors

Thematic behaviors present in wiki small groups between 2014 and 2018	Mean Prevalence (+/− SD)
Learners acquired a conceptual framework for approaching pharmacology as a discipline	100% (+/− 0%)
Faculty provided external guidance that prompted further learning progress	79.5% (+/− 15.0%)
Learners reframed molecular or cellular information to a patient context	69.9% (+/− 17.3%)
Learners established a routine practice of self-directed assessment seeking	68.5% (+/− 14.0%)
Learners asked self-generated questions of one another	63.0% (+/− 17.5%)
Learners identified peer misperceptions or errors and provided gentle correction and guidance	57.5% (+/− 7.0%)
Learners accessed the literature base to provide evidence-based answers	54.8% (+/− 18.3%)
Learners applied their learning at weekly primary care clinical sites (MeSH)	24.6% (+/− 19.2%)

Mean percentage prevalence (+/− standard deviation) of learner responses, that aligned with the thematic behaviors discerned during qualitative analysis, in the 73 wiki small groups that interacted with formative wiki assignments 1–15 and the final, summative wiki assignment between 2014 and 2018

#### Learners established a routine practice of self-directed assessment seeking, across Bloom’s cognitive taxonomy, using the case-based wiki exercises

With respect to health professions education, traditional self-assessment has been defined as a “personal, unguided reflection on performance for the purposes of generating an individually-derived summary of one’s own level of knowledge, skill, and understanding in a particular area.” [41]. Intuitively, learners’ analyses are turned inward; they search themselves as relevant data sources to evaluate and gauge their progress towards competence. However, the validity of this form of self-assessment is often ill-founded and inferences may correspond inaccurately to external standards [42–45]. Instead, we observed externally-oriented feedback requests and invitations consistent with Eva and Regehr’s articulation of self-directed assessment seeking, “the pedagogical process of explicitly seeking external sources of information for formative and summative assessments of one’s current level of performance and practice improvement.” [41]. These behaviors align with initial steps in the self-directed learning process and assist integration of new knowledge [46, 47]. The following passages are representative exemplars of our students’ self-directed assessment seeking behaviors in the wiki assignments.

**Karen**:- I’m wrestling with how to reconcile acetaminophen use in children versus neonates. When the question states that metabolic pathways are reduced in the neonatal period, does that mean that there are less pathways available for the infant to metabolize the drug or that the metabolic pathways occur faster in infants? I read that “the biologic half-life of acetaminophen is somewhat shorter in children and somewhat longer in neonates” (Levy G. Comparative Pharmacokinetics of Aspirin and Acetaminophen. Arch Intern Med. 1981;141(3):279–281). **Mina**, you mentioned that infants are unable to do glucuronidation. In that case, for infants, where the drug elimination is hindered via the lack of a metabolism pathway, wouldn’t more of the drug be shuttled into the other two acetaminophen metabolic pathways? I thought that this would also result in higher levels of the toxic NAPQI intermediate, as the enzyme responsible for detoxifying it (glutathione synthesis) will become saturated with substrate.

- [Wiki #4; Group 13]

**Nasir**:- Would a partial agonist that can exhibit both antagonist and agonist activity have two allosteric binding sites that regulate its function? Or would it just have one binding site and the presence or absence of a ligand determine its response (either agonist or antagonist)?

- [Wiki #8; Group 14]

**Tim**:- Let me know if I overanalyzed this graph. Or analyzed it incorrectly.

- [Wiki #9; Group 13]

**Darrin**:- I wasn’t 100% sure about our answer to question B, so I looked at our drug interaction app on my smartphone and it shows no potential drug interactions identified. If you look at other drugs, like Digoxin, that are transported by P-glycoprotein, strong warnings exist and dosing for drugs reliant on p-glycoprotein is suggested to decrease by 50%. The other thing I would look out for a decrease in the peripheral restriction of opioids and make sure blood concentrations are low.

**Sena**:- That's really interesting, **Darrin**. Nice job thinking to check our drug interaction app! I feel like these things are always more complicated than we know! **Julie** alluded to different processes occurring at different rates with regard to loperamide crossing the blood barrier and being pumped out. I will layer on the glucuronidation process. If this is happening rapidly, yes there will be some loperamide available to cross the BBB, but it will not be in concentrations high enough to exert an effect. Perhaps one of the **faculty** can weigh in on what actually happening? Are we on the right track? :)

**Tim**:- This situation sounds like what we were talking about in Questions 2 and 3. I did some searching and found a site where people posted about their dependence on loperamide as a CNS agent. The discussion board talks about mu receptors, p-glycoprotein inhibitors vs taking very large doses, etc. Interesting because it's relevant to this wiki, but also interesting as an insight into the minds of people struggling with drug dependence, their awareness of the biochemistry, and OTC medications as a target of drug abuse. ***https://www.drugs-forum.com/forum/showthread.php?t=194669***

- [Wiki #12; Group 12]

#### Learners identified peer misperceptions or errors and provided gentle correction and guidance

Peer correction occurs when learners, rather than instructors, point out errors or misunderstandings to one another and help to put them right. It fosters learners’ ability to critically reason and problem solve, and supports the development of soft skills including conveying specific feedback constructively and communicating observations, comments, and actions in a manner that others will attend to and comprehend. Learning how to give and receive peer correction is something that helps prepare health professions students for their future workplace. The following passages are representative exemplars of students’ peer-corrections in the wiki assignments.

**Chloe**:- **Bryan**, I think you may be mistaken. As stated in the question prompt, the volume of distribution of acetaminophen is 57 L, and as stated in the teaching video, the volume of distribution of aspirin is 11 L (not 116 L). I believe you are correct in your second sentence in part however, which indicates that acetaminophen's higher volume of distribution vs aspirin indicates that acetaminophen has a greater capacity to travel beyond the plasma and into the tissues. Great job on part B.

- [Wiki #3; Group 15]

**Karen**:- I think you may have made an error in your calculation for Drug 2. You converted your 1g to 1000mg but in your calculations you used 1g rather than 1000mg.

**Mina**:- You are absolutely right **Karen**! No wonder I was confused by a therapeutic index (TI) of < 1. I have made the corrections.

- [Wiki #8; Group 13]

**Mina**:- Thank you for the thorough explanation, **Evie**. I was having a hard time comprehending the curve before reading your response. Now, I am going to try to take your explanation one step further…

- [Wiki #9; Group 13]

**Jorge**:- Just one other thing to note, **Hannah**, Drug W is an antagonist of H_2_ receptors, not an agonist. So, even if it were to affect H_1_ receptors, it wouldn't cause a runny nose, watery eyes and itching: those symptoms are the result of a functioning histaminergic signaling pathway. Rather, inhibiting H_1_ receptors could induce the nose and eyes to dry out, while also causing drowsiness (assuming Drug W can cross the blood-brain barrier).

- [Wiki #9; Group 9]

#### Learners reframed molecular or cellular information to a patient context

Professional practice requires health professionals to link learning from different sources and disciplines. Here students combined and contextualized various basic science ‘elements’ they encountered in one or more learning experiences in a way that was meaningful at the level of the human patient and relevant to an authentic understanding of what they had learnt. The following passages are representative exemplars of this reframing by students in the wiki assignments.

**Rani**:- I remember not too long-ago seeing commercials for ‘The Purple Pill’ and Tums, each accusing each other of either taking too long to work or not working long enough. Omeprazole is an irreversible antagonist to proton pumps, which inhibits a biological response and takes days for the effect to manifest fully. Tums are made of calcium carbonate and simply raise the pH of the stomach, quickly mitigating the irritation resulting from stomach acid, but not for very long. I wonder if it would be safe to have a patient take Tums while waiting for omeprazole to kick in? They would probably need an easy-to-digest diet (which might help the heartburn in the first place), but would they be at increased risk for GI infection because the pH of the stomach might not be low enough to kill bacteria?

- [Wiki #9; Group 17]

**Lily**:- To link back to my question on patient compliance, I think it is interesting to consider the of patient that would be using a drug like BenGay. This patient would probably be inclined to use a heat pad due to the nature of their symptoms. Certain behaviors that cause adverse effects, such as consuming a certain medication with alcohol, seem less likely to occur together. For example, it seems unlikely that a patient who is sick in bed would also be consuming alcohol. Avoiding alcohol while sick is also fairly common sense. Patients taking medication like BenGay may unknowingly fail to comply just because they are doing what seems innocuous and common to treat their symptoms.

- [Wiki #10; Group 16]

**Elias**:- I thought that **Darrin** gave great answers. Going along with part a, I was reading about opioid overdose in an article published in the NEJM. It stated that respiratory depression (of 12 breaths or less a minute in a not sleeping patient) was one of the best signs of overdose, especially if it is coupled with constricted pupils or stupor. **Darrin’s** answer about the lethargy/sedation made me think of this. Here is the article if you want to read it. ***http://www.ncbi.nlm.nih.gov/pmc/articles/PMC3739053/***

- [Wiki #12; Group 12]

#### Learners applied their learning at weekly primary care clinical sites (MeSH)

The pre-clerkship phase of the FHN SOM curriculum consists of three courses: Foundations of Medicine (FOM), Clinical Arts and Sciences (CAS), and Scholarly Reflection and Concentration Capstone (SRCC). CAS includes extensive small group experiential learning with standardized patients, video review, goal setting, and clinician preceptor feedback. In addition, each first- and second-year medical student is paired with a practicing community physician, and spends 1 day per week at either an internal medicine, pediatric, or family medicine site learning from their physician mentor (medical student home; MeSH). Under supervision, students are able to practice essential clinical skills with patients, including interviewing and examining patients, engaging in clinical reasoning skills and the diagnostic process, and assisting in creating treatment plans. The following passages are representative exemplars of how students’ applied their pharmacology learning at their weekly MeSH sites in the wiki assignments.

**Robyn**:- I agree that dose is harder to conceptualize when the medication is a cream instead of a pill. I wonder if this would be a benefit of transdermal patches since each patch is a dose (though these are often for systemic effects not just local effects). Then again, I think the idea of a drug getting absorbed through the skin and having the desired systemic effect on the body may be hard for some patients to understand. At **MeSH** today, there was a patient who told me that she used nicotine patches but they didn't stick that well so sometimes she just stuck another one on. She wasn't sure if this was okay or if she was getting a double dose.

- [Wiki #10; Group 7]

**Calum**:- I just wanted to add to **Dr. McHugh's** point about addiction. Nicotine has a half-life of about 40 minutes, which explains why many people smoke a pack of cigarettes/day as it keeps their levels of nicotine relatively constant. With that said, chewing tobacco or snuff allow for easier use and therefore increase the likelihood of developing an addiction. I was able to talk about this with a patient at **MeSH** this week who had begun to use chewing tobacco while at work as part of his efforts to quit smoking; it was very easy for them to sit at their desks for hours with a ‘lip’. He had thought that only inhaling tobacco smoke led to nicotine addiction.

- [Wiki #13; Group 9]

**Hannah**:- I was discussing with my **MeSH** preceptor this week how to counsel a 16-year-old patient who had asked about quitting smokeless tobacco use on his baseball team. As Jacob's student physician I wanted him to quit. But I also wanted to be careful in counseling him. I was worried that, as a 16-yr-old, he might shut down and not listen, perceiving a need to be completely independent. I discussed with him the harmful effects of tobacco in all its forms, and showed him pictures of what lungs of smokers look like. We discussed him being on the baseball team and many of his teammates are chewing tobacco, so it has been hard for him to say no…I think it was important to listen to him and not just lecture him on what he should or should not be doing.

- [Wiki #13; Group 9]

#### Learners accessed the literature base to provide evidence-based answers

The continually expanding availability of biomedical information means that one-time knowledge learning (i.e., only while in medical school) is insufficient to deliver optimal patient care. Physicians must instead routinely gather, appraise, and interpret medical evidence in order to apply knowledge at the point of care [48]. Here learners cited pertinent information using credible and varied evidentiary sources. The following passages are representative exemplars of how students’ accessed and cited peer-reviewed literature in the wiki assignments.

**Tim** - I found this PubMed article on the beverage chosen during administration of aspirin***.*** **Odou P, Barthélémy C, Robert H. Influence of seven beverages on salicylate disposition in humans. J Clin Pharm Ther. 2001;26(3):187-193.** ***http://www.ncbi.nlm.nih.gov/pubmed/11422602*** It can be viewed using the library PubMed link. Some interesting factors that were brought up were...1) length of stay in stomach (due to food stuffs in orange juice slowing down stomach evacuation into the intestine; 2) solubility of aspirin in the more acidic orange juice (less soluble in strongly acidic environments, more soluble when ionized in less acidic environments). It's a catch-22 because the less soluble form of aspirin is also the more membrane permeable form that can enter the blood stream from the stomach.

- [Wiki #1; Group 8]

**Luke**:- I found an article from the Journal on Studies on Alcohol that related the intake of food to the enzymatic metabolism of alcohol. The authors used Michaelis-Menton Kinetics to demonstrate this fact. They also indicated that not only is the absorption of alcohol slowed, but the highest concentration of alcohol within the blood stream is lowered with an intake of food. Lastly, it takes a longer amount of time for alcohol to reach its peak concentration with food consumption. ***A.J. Sedman, P.K Wilkinson, E. Sakmar, D.J. Weidler, and J.G. Wagner. "Food Effects on Absorption and Metabolism of Alcohol." Journal on Studies on Alcohol 37.9 (1976): 1197-214. PubMed. Web. 14 Aug. 2014***

- [Wiki #1; Group 8]

**Camden**:- The use of a heating pad can dramatically increase the amount of methyl salicylate absorbed through topical administration. **Cooper (2007). How can you overdose on BenGay?** ***http://scienceline.org/2007/08/ask-cooper-bengaydeath/***

**Andrés**:- In addition to the factors mentioned by **Camden**, heat also causes local vasodilation and increased vascular permeability, thus higher-than-intended and faster-than-intended concentrations of the topical drug in systemic circulation. The mechanisms for this vasodilation include “the roles of temperature-sensitive afferent neurons as well as nitric oxide” (***Kellogg, In vivo mechanisms of cutaneous vasodilation and vasoconstriction in humans during thermoregulatory challenges, J Appl Physiol 100: 1709 –1718, 2006).***

- [Wiki #10; Group 16]

**Sena**:- I found an interesting article looking into whether bacteria in the gut produce GABA, and possible implications for inflammatory diseases of the bowel as well as GABAs involvement in pain signaling in the gut. **American Society for Microbiology (2012). Intestinal bacteria produce neurotransmitter, could play role in inflammation.** ***http://www.eurekalert.org/pub_releases/2012-06/asfm-ibp061312.php***

- [Wiki #11; Group 12]

**Evie**:- I found two papers showing that nicotine increases the permeability of the Blood-Brain Barrier, which I thought was interesting. The first paper showed that more sucrose crossed the BBB with continuous administration of nicotine. The second paper reproduced that result, and also measured a decreased in MLA uptake, a selective antagonist of the nicotinic ACh receptor alpha7. This could have an impact on patients with chronic nicotine use, as they may be more affected by exposure over time**. Hawkins et al. (2004).** ***Brain Res. 2004 Nov 19;1027(1-2):48-58;*** **Lockman et al. (2005).** ***J Neurochem. 2005 Jul;94(1):37-44.***

- [Wiki #11; Group 13]

#### Learners asked self-generated questions of one another

Effective teaching requires that learners not be mere receptacles of knowledge but rather they should participate meaningfully and actively in their own learning processes. One example of this is students generating questions for themselves to enhance comprehension [49]. The following passages are representative exemplars of students’ asking self-generated questions in the wiki assignments.

**Chloe**:- I agree with your responses **Robbie**. Here are some questions of my own. Do antihistamines specifically inhibit the release of epinephrine and norepinephrine that subsequently contributes to the drowsiness effect of antihistamines? I have heard that non-drowsy antihistamines have caffeine added to them to counteract the drowsiness classically associated with antihistamines. Is this true, or are non-drowsy antihistamines formulated in such a way that they physiologically (through specific receptor binding) do not cause drowsiness?

- [Wiki #7; Group 15]

**Lily**:- I understand that increasing the dose would increase the concentration in a particular body compartment. In terms of lethal dosage, is this dependent on which compartment is most sensitive to the drug? For example, the concentration in one compartment could be lethal, but not in others.

- [Wiki #3; Group 16]

**Mitchell**:- Are there situations in adulthood where metabolic pathways are reduced as in the neonatal period? I know that some adults don't process acetaminophen (paracetamol) properly or have an adverse reaction to it, and I am wondering if the underlying mechanism could be the same as that present in a neonatal infant?

- [Wiki #4; Group 18]

**Ria**:- I have a question: Are Phase I enzyme polymorphisms associated with Phase II enzyme polymorphisms? Like a very rapid alcohol dehydrogenase activity polymorphism associated with a low-to-no activity acetaldehyde dehydrogenase polymorphism?

- [Wiki #5; Group 17]

#### Faculty provided external guidance that prompted further learning progress

Giving learners feedback to guide their progress has always been viewed as beneficial to learning; however, the need to combine opportunities for learner reflection and coaching with feedback to help learners achieve anticipated outcomes has been under-stressed [50]. In a learner-centered coaching approach, teachers use available formative performance data to contribute specific feedback that is relevant to fostering continued momentum towards mutually discernible instructional targets. The following passages are representative exemplars of faculty providing external guidance to prompt further learning progress in the wiki assignments.

**Dr. McHugh:** Strong work, **Ahmed**. Would anyone like to comment on volume of distribution (V_D_) and whether we would want a drug with a high V_D_ or a low V_D_ administered to treat a patient with bacteremia? And why?

**Rani**:- Bacteremia is the presence of bacteria in the blood. To effectively combat the infection, the (presumably) antibiotic should remain at the site of infection. This means the drug should have a low V_D_. It won't distribute to the body compartments but instead remains in the plasma where it has the best chance of fighting the infection.

- [Wiki #3; Group 17]

**Dr. Macica**:- **Padme**, I'm glad you're thinking about the impact of blood flow (and tissue extraction rate); does anyone want to comment on how this helps inform the question about Mrs. N and the synovial joint ?

- [Wiki #3; Group 14]

**Dr. Hall**:- **Yasin**, Can you clarify a bit? C0 is a *derived* value. The drug distributes quickly and we can't really ‘catch’ the concentration of drug at time=0. How do we extrapolate from your sampling data to obtain C0?

**Yasin**:- We could use the data from the blood samplings to plot the change in dose over time. We could then use the plot to extrapolate the exact dosage at time = 0 by utilizing a curve that best fits the data. Fitting the plot to a curve does not yield the Co value- you can see from the video that the curve is extrapolate back to t=0 to obtain an estimate for C0.

- [Wiki #3; Group 4]

**Dr. McHugh**:- What do you folks think about the fact that we are talking about OTC drugs here, and that there will be many people taking them who really have different alleles for CYP2D6? Do you that as the availability of individual genetic testing increases in the future, people will want to know about their CYP450 profile? Or do you anticipate people will be scared of genetic profiling information?

**Ria**:- I think that to a certain degree there will be self-selection of how people choose to take OTC medications. I know that I have a funny reaction to diphenhydramine unless I take it under specific circumstances, and I know others who refuse to take other kinds of OTC medication because of the reactions they've had. It's very likely that these odd reactions could be based on genetic polymorphisms, but we didn't have to do genetic testing to find that out. I think people will certainly want to know their genetic profile, out of curiosity or medical concern or both. The popularity of genetic sequencing and interpretation services (e.g., 23 And Me) speaks to that desire. However, there are two downsides to gaining that knowledge: 1) Having that knowledge of the near-certainty of things that may happen to you (if there are indeed nasty surprises lurking in your DNA), and 2) health insurance companies knowing the same thing. Everything is a risk-reward analysis, and I think it should be left up to individuals to make the decision to access that knowledge.

- [Wiki #5; Group 17]

**Dr. McHugh**:- Are you confident that folic acid can diffuse through cell membranes at pH 6? Despite the +1 of the amino group and the -1 of the carboxylic acid canceling each other out in the overall sense, you still have a molecule that has two charged functional groups (i.e., a zwitterion). Do you folks think there is a difference between 'neutral' and 'uncharged' when it comes to crossing the plasma membrane. Module #1's teaching video outlined 4 main ways by which small molecules cross cell membranes.

- [Wiki #2; Group 14]

## Discussion

The social pedagogy curricular model we developed, in which pharmacology learning was nested, birthed a culture embraced and sustained by our community of teachers and learners. This culture involved processes where learners and faculty, over the course of the academic year, acquired habits of self-directed assessment seeking and learner-centered coaching, respectively [3, 41]. In other words, the model taught learners to look outward to peers, faculty, and external sources of information for credible and constructive feedback, and that this feedback could be trusted as a basis to direct performance improvement [39]. Our approach represents an attractive alternative to lecture- or PBL-based learning formats, and is well-placed to lessen students’ reliance on internal, unguided thought processes to gauge the adequacy of their knowledge, skills, and understanding.

### Expected outcomes, learner assessment, & helpful feedback

A primary expected outcome was that by the end of the curriculum learners would have acquired a conceptual framework for approaching the discipline of pharmacology. We drew upon existing scholarship to inform our definition and operationalization of ‘conceptual framework’ as a measurable variable of interest. Put simply, a concept is a term for a generalization. It may label or symbolize objects with real, material existence (e.g., fruit, machines, winter clothing) or abstract ideas and knowledge domains (e.g., civility, scorn, humor). Concepts may originate from experience or by modifying existing ideas. That said, a conceptual framework is not simply a haphazard arrangement of abstractions, ideas, or specialized categories – but, rather, a holistic *construct*. We functionally defined a conceptual framework as, “a network of interlinked concepts where intersecting, related abstractions provide an interpretive context that facilitates comprehension, analysis, and explanation of observed phenomena” [38, 40, 51, 52]. In line with this, our learners employed an array of conceptual framework elements as a scheme for choosing and prioritizing salient variables, and providing coherence between their appraisal of pertinent pharmacological data and eventual conclusions. Thus, the final individual wiki assignments served as the link between summative judgement of competence and the conceptual framework construct. They offered learners the opportunity to demonstrate cognitive flexibility through using their conceptual framework to evaluate and interact competently with unfamiliar drug information. This represents a meaningful step towards the development of adaptive mastery and away from fact memorization and recall.

Miller stated that “no single method of assessment could encompass the intricacies and complexities of medical practice.” [53]. He argued that we should use methods that elicit evidence appropriate to the degree we wish learners to perform competently. Miller’s ideas focus on educational outputs, recognizing that teaching is not the same as learning. At the culmination of learning processes our attention should be directed towards what learners can do, not what has been taught to them. This rationale underpinned the choice of case-based wiki exercises as an assessment strategy that repeatedly crisscrossed Bloom’s cognitive taxonomy. This formative assessment plan is in line with Duncan-Hewitt’s recommendation of giving students numerous variations of drug-related problems, and opportunities to solve them, in order for deep learning to occur [54]. The recurrent intersection of each small group of five learners with case-based questions, anchored to multiple tiers of Bloom’s scheme, permitted faculty to challenge students’ mastery of pharmacokinetics and pharmacodynamics and provide ongoing timely feedback.

The Blackboard® learning management software provided technological means for pharmacology faculty to monitor unobtrusively asynchronous learning that occurred from the wiki small groups’ interactions with the case-based assignments. This technology facilitated faculty’s ability to weave in formative assessment during independent learning: we were able to query their mastery across Bloom’s taxonomy, ask them to commit to a short answer, and then see the response. It represents a low-key, but informative system for collecting data on learner outputs, in order to monitor how well they align with salient competencies, over the period of pharmacology learning [55]. Our arrangement demonstrates how learner-centered coaching and helpful feedback can be embedded into the infrastructure of the learning environment in support of Lefroy et al.’s helpful feedback motif [7].

### Consistency of fit with invoked theories

Having reflected on the literature and DM’s experience of teaching pharmacology, *constructivism*, *communities of practice theory*, and *networked learning theory* were consciously chosen as current, heuristically interesting models that articulated mechanisms and principles of learning pertaining to the perceived need for learned-centered coaching and helpful feedback. These three educational theories served as a lens to guide the curricular design and data analysis. The observed behaviors, processes, outputs, and outcomes resulting from our social pedagogy model were largely consistent with those anticipated by the invoked theories. For example, formative assessment as a supportive conversation between pharmacology subject matter experts and the constituents of each wiki small group upholds the tenets of communities of practice theory and networked learning theory, and fosters cognitive bootstrapping (i.e., moving learners from well-defined to complex problems), scaffolding (i.e., supporting learners at the beginning of problem-solving and gradually removing the structure), and flexibility (i.e., the ability to internalize how to solve variations of a situation independently in the future) [16, 56]. One phenomenon not predicted was that of some wiki small groups to engage in an abbreviated form of *streaking*. In social media terms, streaking is when participants communicate back and forth for several consecutive days [57]. With us, it took the form of small group members continuing to refine their knowledge and comprehension by building consecutively upon one another’s posts for a prolonged time interval. The ‘relatedness’ dimension of self-determination theory may be relevant in explaining the motivation of this behaviour in some groups but not others [58]. In other words, the degree to which learners perceive relationships and interactions with their group co-members as personally meaningful may influence their drive to contribute. Alternative explanations could be crossover behaviour from social media use or overcompetitive ‘gunner’ comportment [59]. Further investigations are required to test these hypotheses.

### Sustainability and threats to validity

This social pedagogy approach to teaching pharmacology has been sustained at our institution with a high degree of acceptance by learners and faculty. Based on the Blackboard® access metrics for 2014–2018, learners and faculty alike participated in the curriculum across the M1 academic year at the designated time points. We found implementation to be low-cost in terms of $ amount; we had all the material resources required with the exception of video production software. In terms of time, investment was more than that required for creating a lecture but less than that for developing a PBL case and training PBL facilitators. We found the time needed to develop each teaching video to be equivalent to that for a 1-h lecture; likewise, for the wiki assignments and example answers. In addition, the pharmacology faculty spent approximately 2 h per module monitoring, assessing, and responding to wiki assignments. To ensure sustainability of the approach from a faculty perspective, it is important to recognize the contribution that faculty members make in teaching this curriculum. Otherwise, their commitment to teaching, assessment, and guidance to learners may go underappreciated given the asynchronous, online format of the model. Of note, evaluation comments from a few students suggest that some participants may perceive lecture and passive pedagogies to be more effective if they are not familiar with processes of learning and rely on only themselves to form a judgment on the efficacy or suitability of a particular pedagogical strategy [41]. This echoes previous pharmacology-focused and other basic science reports where students’ perceptions of active learning methods tended to be negative regardless of improved learning outcomes [60, 61].

We anticipated four potential threats to validity (i.e., sources of error) that our system of assessment may be vulnerable to: (1) non-response, (2) inter-rater variance, (3) learners looking for ‘answers’ online, and (4) plagiarism. Steps to reduce these should be considered by anyone contemplating adoption of this approach to learning. *Non-response* was not an option for our learners as the social pedagogy-based pharmacology curriculum is a required course element of ‘Foundations of Medicine I’. With regard to *inter-rater variance*, the steps faculty took to mitigate this potential threat to validity included calculating inter-rater agreement from a subsample of wiki assignments with a ‘pass’ interpretation, developing an explicitly clear rubric and set of example answers, and a policy of mutual review and consensus agreement regarding an interpretation of ‘fail’ for any particular individual’s assignment output. In terms of *learners looking for ‘answers’ online*, with access to a digital world of ubiquitous information sources, we anticipated that students would turn to the internet to seek and gather additional information as needed. This is consistent with networked learning theory and self-directed assessment seeking [41], and may represent a learning opportunity rather than a validity threat. The ability to search, screen, select, and evaluate relevant information sources then extract pertinent details before synthesizing them into an original exposition is another evidentiary facet of the presence of a conceptual framework operating as an interpretative lens crisscrossing Bloom’s cognitive taxonomy. *Plagiarism* is most likely to threaten validity if expressed in the form of ‘forward feeding’ of completed wiki assignments from one class of learners to the next. For example, via a shared Dropbox® of ‘supportive’ resources. Ideally, an institutional subscription to anti-plagiarism software would identify any occurrence of this problem (or generic plagiarism). Alternative strategies include maintaining a complete record of all previous cohorts’ wiki assignments, maintaining an attitude of vigilance for any written response suspected of not being original, and discussing this openly with each new class of students in the context of professionalism and an institution’s honor code.

### Strengths and limitations

This investigation examined the feasibility of developing, implementing, and sustaining an innovative model of social pedagogy, within which pharmacology learning was nested. Its qualitative methodology allowed for rich exploration of 4 years of learner and teacher co-participation; however, it also could have benefited from the perspective of students from other institutions. Given our abductive approach to data analysis, we must acknowledge that our interpretations may be plausible yet off-target. Learners had supervision from physician mentors at weekly primary care clinical sites (MeSH) who were positioned to potentially assess and comment on the students’ ability to reason with regard to therapeutics. Such data are relevant to physicians-in-training developing the skillset to prescribe rationally. Unfortunately, it is not something we had the forethought to assess and collect data for. We believe that retrospectively seeking such data now would suffer unavoidably from recall bias; however, it is something we would like to include in a next-steps investigation of the curriculum’s impact. Future studies should focus on examining the impact of this social pedagogy model: (1) when co-present with other learning modalities (e.g., PBL), and (2) downstream in the clinical phases of medical education curricula.

### Implications for medical education

Medical education continues to be a recurrent locus of earnest calls to respond to a variety of pressing challenges. To name a few: the increase in medically-related information, the ubiquity of mobile technologies that allow access to this information, changes in healthcare delivery models, the evolving role of the patient who has more information about their illnesses than ever before, and conspicuous healthcare gaps and inequalities [62–64]. As part of their global independent commission report on how to implement changes that will improve health systems, Frenk et al. proposed three necessary pivots: “1) from fact memorization to searching, analysis, and synthesis of information for decision-making; 2) from seeking professional credentials to achieving core competencies for effective teamwork in health systems; and (3) from non-critical adoption of ‘orthodox’ pedagogies to intentional selection of evidence-based educational models that can be creatively adapted to address local priorities.” [62]. Information transmission-oriented and problem-based learning are two such ‘orthodox’ pedagogies that have challenges and fall short of their fullest potential [9]. In a pharmacology learning context, two common difficulties they may induce are the tendency of learners not to ask higher-level questions and the potential for incongruency between self-directed learning and external expert guidance. In PBL, learners purposefully receive limited feedback and coaching from faculty; this is largely due to self-directed learning being a central tenant of PBL [18]. Effective, rather than arbitrary, self-directed learning entails the possession of some baseline, referential competence; it requires that the learner know the extent and scope of their own learning gaps, when their efforts are sufficient to address them, and when to dig deeper and strive further [20]. Pre-clerkship medical students may not possess this prerequisite competence given their unfamiliarity with the subject matter, which may blind them to knowledge and comprehension deficits [20]. Inadequate experience with pharmacology may weaken and compromise their self-assessment processes in the absence of sufficient external counsel [20]. Our social pedagogy-based model addresses these two common difficulties by providing opportunities to practice and reflect upon higher-order thinking that iteratively crisscrosses Bloom’s cognitive taxonomy; and for the emergence of self-directed feedback seeking and sharing from peers and faculty experts. This occurs in a context that embraces asynchronous internet-based technologies, the ubiquity of medically-relevant information, and participatory media as a means for healthcare professionals in-training to learn and interact together.

## Conclusion

We instituted a theory-informed social pedagogy-based curriculum for teaching pharmacology to pre-clinical medical students. Our approach has implications as part of the larger trending reform movement in health professions education that targets the processes and skills of thinking and the ability to adapt to situations as much as the knowledge base of learners. This learning model yielded many benefits, including: (1) instilling habits of effective teamwork and shared responsibility early; (2) allowing learners to acquire a conceptual framework that provides an interpretative context for comprehension, analysis, and synthesis of drug information instead of fact memorization and recall; and (3) creating a supportive learning environment where learners, across an academic year, acquired a routine practice of self-directed assessment seeking that clarified and supported leaners’ awareness of their developing competencies, challenged them to strive for improvement, and enhanced their self-efficacy. This social pedagogy model is agnostic with regard to pharmacology and type of health professional learner; therefore, we anticipate the model to be compatible with, and these benefits transferable to, other disciplines.

## Supplementary Information

**Additional file 1.** Distribution of online pharmacology modules in relation to first year teaching blocks, elements of the conceptual framework being introduced, and over-the-counter drug context.

**Additional file 2.** Example small group wiki assignment.

**Additional file 3.** Small group pharmacology wiki grading rubric.

**Additional file 4.** Representative example of a final, summative wiki assessment submission.

**Additional file 5.** Summative Pharmacology MCQ Item Statistics.

**Additional file 6.** Percentage of students who failed formative small group wiki assignments and the percentage of failures due to timeliness, quality or authorship/citations/respect for M1 students 2014–2018.

## Data Availability

All data generated or analysed during this study are included in this published article [and its supplementary information files].

## Abbreviations

AY: Andrew J. Yanik
CAS: Clinical Arts & Sciences
CoP: Communities of Practice
DM: Douglas McHugh
FHN SOM: Frank H. Netter MD School of Medicine
FOM: Foundations of Medicine
IRB: Institutional Review Board
M1: First-Year Medical Students
M2: Second-Year Medical Student
MCQs: Multiple-Choice Questions
MeSH: Medical Student Home
MD: Medical Degree
MM: Michael R. Mancini
OTC: Over-The-Counter
PBL: Problem-Based Learning
SRCC: Scholarly Reflection and Concentration/Capstone

## Footnotes

1 This statement is grounded in: 1) conversations between DM and his network of pharmacology educator colleagues and their contacts in North America, Europe, and Oceania, as well as his experience attending meetings and conferences of the British Pharmacological Society (BPS) and American Society of Pharmacology and Experimental Therapeutics (ASPET) from 2003 to 2020; and 2) the work of Kincade S. (2005) that examined the prevalence of PBL in the preclinical curricula of the 123 Liaison Committee on Medical Education-accredited medical schools in the United States between 2003 and 2004 (the number of accredited schools has subsequently expanded). Kincade found there is wide variation in PBL implementation across medical schools, but at least 109 U.S. medical schools have used this methodology to some extent, and 37 schools have relied on PBL as their major teaching method. Kincade S. A snapshot of the status of problem-based learning in U.S. medical schools, 2003–04. Acad Med. 2005;80:300–301.
