# Supplementary material for: An innovative pharmacology curriculum for medical students: promoting higher order cognition, learner-centered coaching, and constructive feedback through a social pedagogy framework
Source: BMC Med Educ. 2021 Feb 5;21:90. doi: 10.1186/s12909-021-02516-y (PMC7863331; doi:10.1186/s12909-021-02516-y)

**Additional file 1:** Distribution of online pharmacology modules in relation to first year teaching blocks, elements of the conceptual framework being introduced, and over-the-counter drug context.


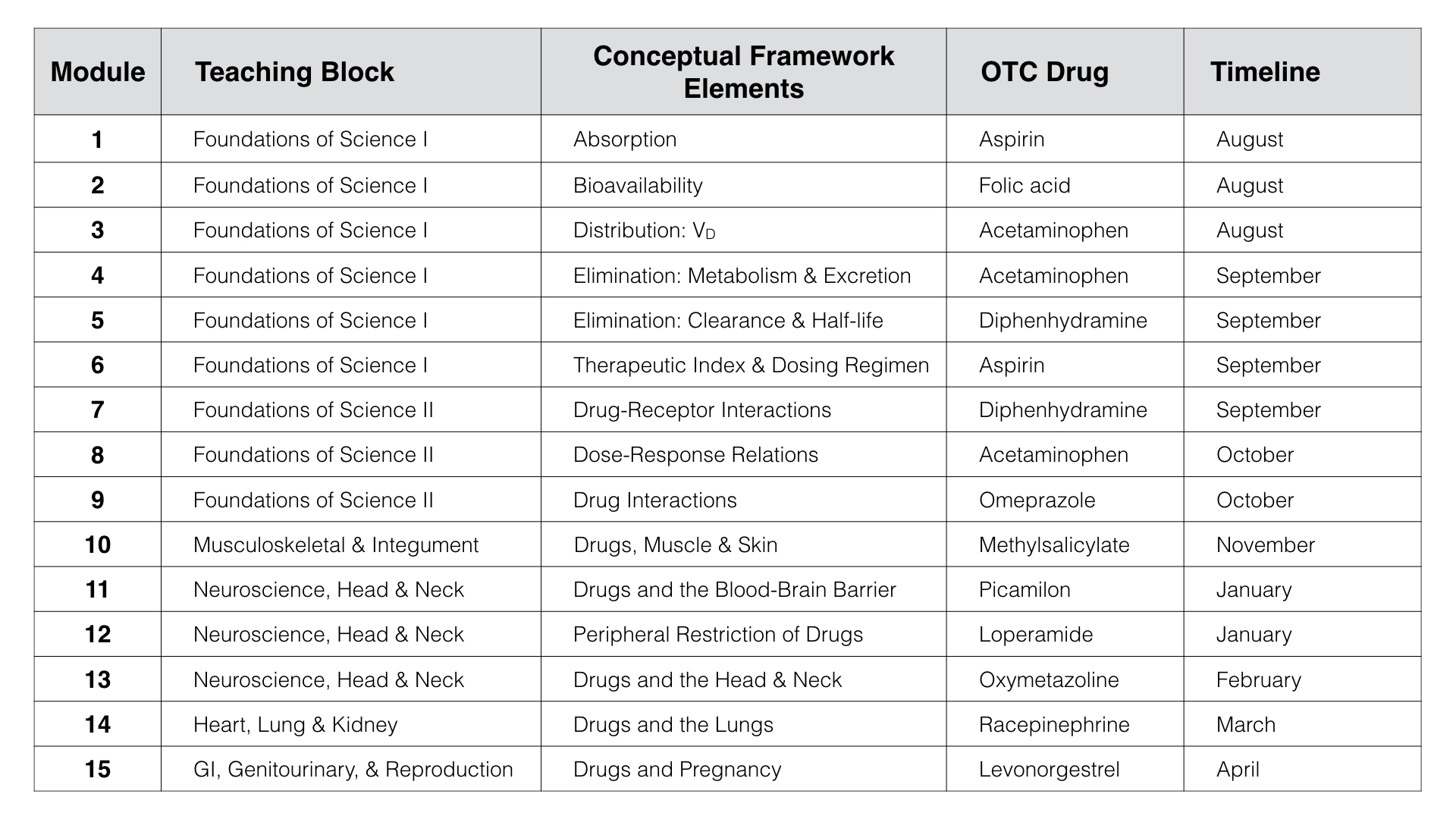

Supplement: Supplementary file 1 — Additional file 1. Distribution of online pharmacology modules in relation to first year teaching blocks, elements of the conceptual framework being introduced, and over-the-counter drug context. [file 12909_2021_2516_MOESM1_ESM.docx]
