# Supplementary material for: An innovative pharmacology curriculum for medical students: promoting higher order cognition, learner-centered coaching, and constructive feedback through a social pedagogy framework
Source: BMC Med Educ. 2021 Feb 5;21:90. doi: 10.1186/s12909-021-02516-y (PMC7863331; doi:10.1186/s12909-021-02516-y)
Supplement: Supplementary file 2 — Additional file 2. Example small group wiki assignment. [file 12909_2021_2516_MOESM2_ESM.docx]

**Additional file 2:** Example small group wiki assignment


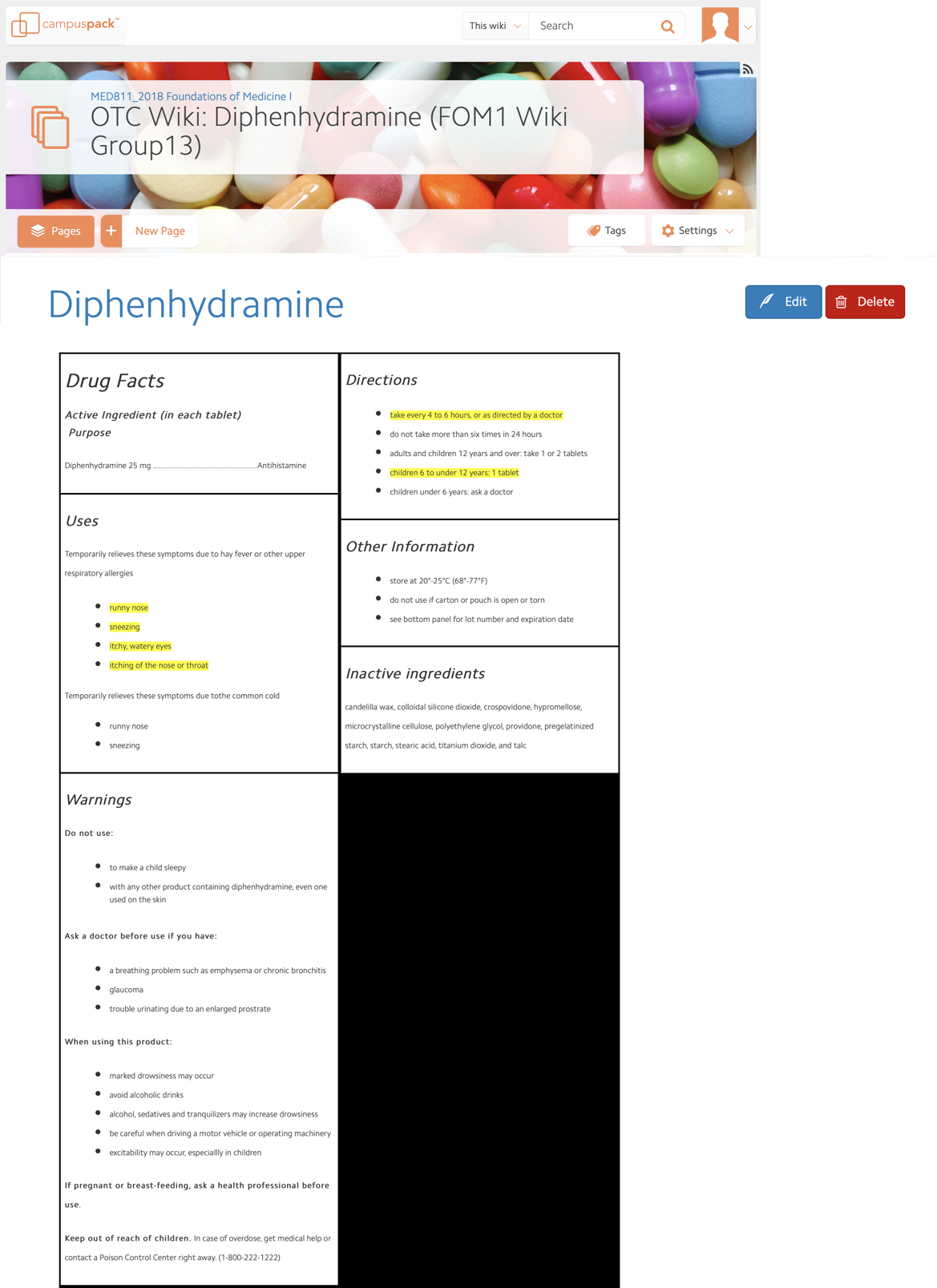


***Case Vignettes***

**
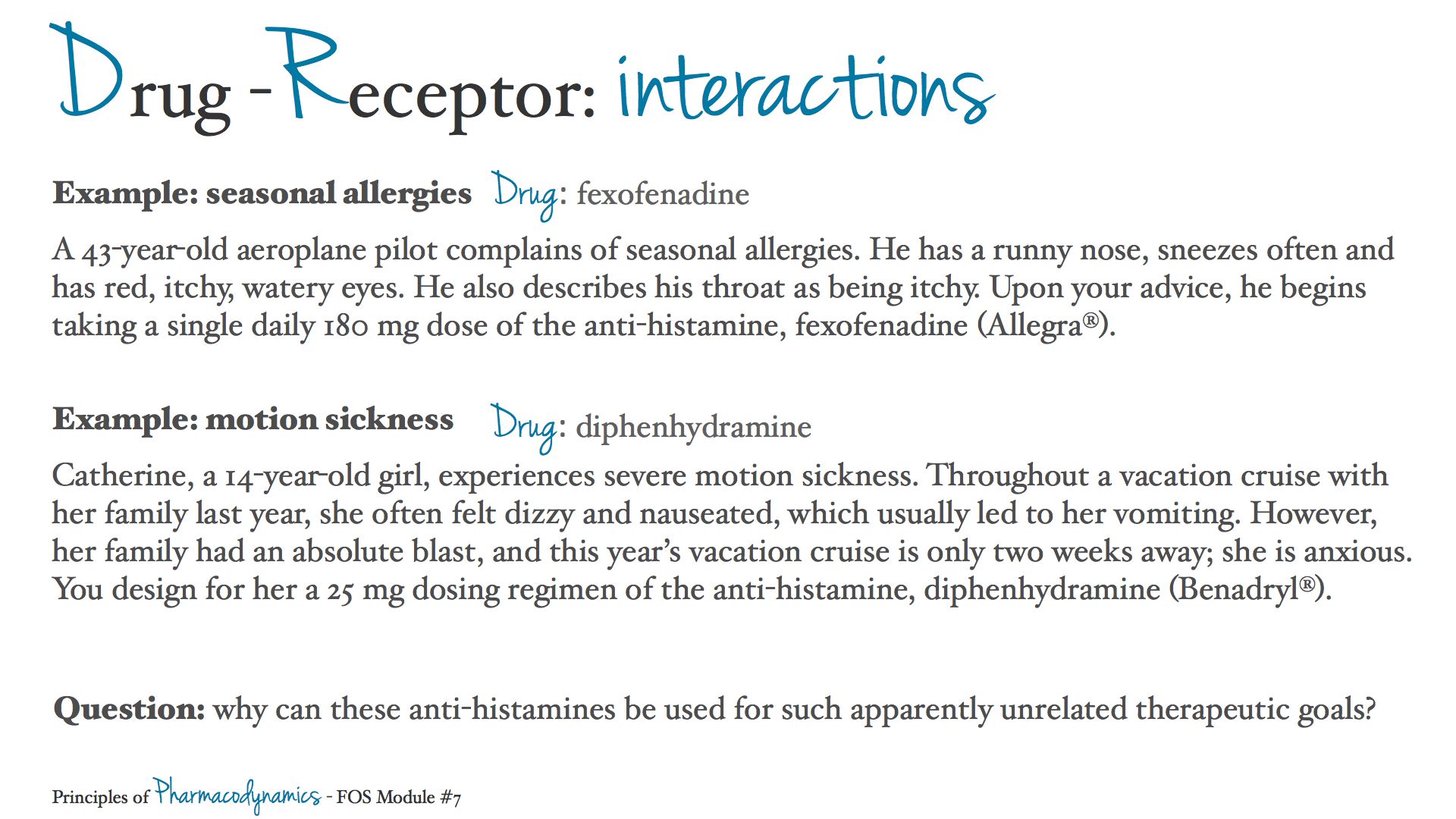
**

***Question 1***

*a. Which histamine receptor subtypes are responsible for producing the symptoms highlighted in the drugs facts above?*

**Sena** :- Some of the key allergy/cold symptoms highlighted above include sneezing, itchy watery eyes, runny nose, and itching of the nose and throat. Histamine increases the permeability of capillaries allowing more plasma to leak out presenting in the form or watery eyes and runny nose. Histamine binds to G-protein coupled receptors (GPCRs) to exert its physiological effects. There are four histamine GPCR subtypes: H_1_, H_2_, H_3_, and H_4_. The Histamine-H_1_ complex is responsible for increased capillary permeability and stimulating sensory nerve endings that mediate pain and itching.

*b. What type of signal transduction mechanism is involved?*

**Sena** :- Histamine binds to GPCRs which utilize a second messenger system because they are membrane proteins receptors. The intracellular surface of the receptor is where the trimeric G-protein binds the receptor. A ligand which is histamine in this case, binds to  GPCR and triggers the release of the alpha subunit of the G-protein. The alpha subunit is a GDP carrier which is exchanged for a GTP by GDP-GTP exchange factors. The activated (GTP carrier)alpha subunit then binds to adenylyl cyclase (AC) which is another integral membrane protein. The GTP-AC complex can convert ATP to cAMP, a secondary messenger, which can then activate effectors molecules such as Protein Kinase A. This signal transduction mechanism is useful for hydrophilic molecules (i.e. histamine) that cannot cross the phospholipid membrane to exert influences on intracellular compartments and chemicals.

**Faculty Preceptor** :- What specific G protein alpha subunit and second messenger signaling is involved?

**Karen** :- I think you may have H_1_ and H_2_ switched. The specific G protein alpha subunit in H_1_ receptors is the Gq subunit which signals via activating phospholipase C. Phospholipase C acts on membrane lipid, phosphatidylinositol and cleaves PIP_2_ into IP_3_ and DAG. *Similar to the alpha1-adrenergic receptors*. IP_3_ can bind to a receptor on the ER and cause the release of Ca^2+^, and DAG in association with Ca^2+^ activate Protein Kinase C. H_2_ receptors cause the increase in cAMP via Adenyl Cyclase.

*c. Explain why diphenhydramine is inappropriate for treating the pilot’s seasonal allergies (see case vignettes above).*

**Sena** :- Diphenhydramine is inappropriate for treating the pilot's allergies because it is an H_1_-selective antagonist. While histamine binding to H_2_ receptors can trigger the itchiness and watery eyes and nose associated with seasonal allergies, in the CNS, this binding is excitatory and maintains arousal. This explains why some anti-histamines have sedative effects. If the pilot were to take diphenhydramine, his itchy and watery eyes and runny nose would stop, but he would also not be able to stay awake, which poses a rather large issue for someone operating an airplane - especially if it is a long flight. Histamine also works to process sensory input in the CNS to stabilize balance and spatial coordination. If histamine cannot bind to H_1_ receptors in the CNS, it cannot maintain a vestibular balance and coordination. hand-eye coordination, spatial anticipation, and balance are 3 traits that are imperative for a pilot to have. Antihistamines such as diphenhydramine that bind the H_1_ receptors would jeopardize these skills.

**Faculty Preceptor** :- Your claim regarding H_2_ receptors contradict what you said in part a about H_1_receptors being responsible for the production of seasonal allergy symptoms. It might be a good idea to watch that section of the teaching video again. It will help clarify the vagueness of part b too.

**Eva** :- Diphenhydramine, more commonly referred to by the brand name Benadryl, is a bad idea to take if you need to fly a plane. As Sena said, it crosses the blood/brain barrier and exerts effects on the CNS. In addition to treating allergies, diphenhydramine is also used as a sleep aid. A more appropriate treatment might be fexofenadine, which only binds to peripheral H_1_ receptors.

***Question 2***

*Catherine’s cruise ship will launch in 2 weeks' time (see case vignettes above). Assume the prophylactic (preventive) benefits of diphenhydramine require her to reach plasma concentrations within the therapeutic window.*

*a. How many half-lives are required for drug accumulation to reach 90% of Css?*

**Karen** :- According to Wiki Assignment #6, 90% of the steady-state drug concentration is achieved in 3.3 half-lives when the drug doses are repeated or given continuously.


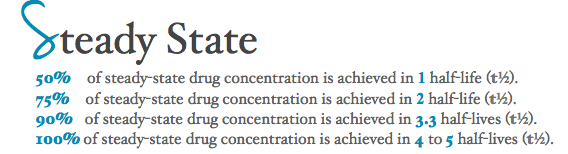


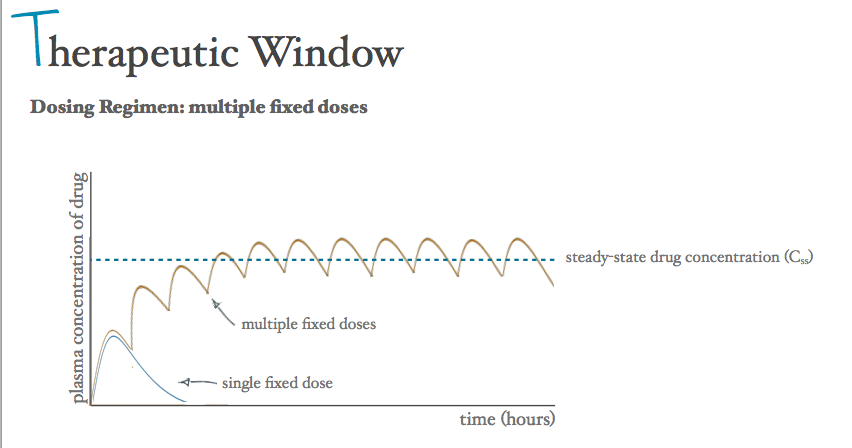


*b. If diphenhydramine has a half-life of 7 hours, and the cruise ship launches at 3pm, approximately when should she start taking the drug?*

**Karen** :- Catherine needs to take the drug early enough to reach a concentration within the therapeutic window prior to the launch of the cruise ship. Since we were not given a therapeutic window for diphenhydramine, I will assume that at minimum we want a 90% Css in my calculations. To determine how soon Catherine should start taking the drug you would first want to determine the number of half-lives needed to reach that concentration. From part a, we determined that 3.3 half-lives should allow the drug to reach the needed 90% Css level in the plasma. Since diphenhydramine has a half-life of 7 hours, this would mean that at least (7hrs x 3.3= 23.1 hrs) 23.1 hours of her maintaining her drug dosing regimen would be needed to get to the proper steady state plasma concentration of the drug. Therefore, I would recommend that Catherine begin taking the prescribed dosage of drug 24 hours before her boat launch (* 3pm the day before) and continue with the recommended 25 mg dose every 4-6 hours throughout the course of her trip.

**Justin** :- Great answer Karen. The instructions for diphenhydramine state to not take the drug more than six times in 24 hours, so if Catherine takes it every 4-6 hours that is okay. If she takes diphenhydramine more than that, it could potentially reach toxic levels in her blood. Additionally, even though the drug will help relieve her dizziness and nausea, there are other side effects she should be aware of. Tiredness, sleepiness, and disturbed coordination can occur. These may be side-effects that can negatively affect her vacation.

***Question 3***

You are involved in a research study that is generating novel drugs targeted at the histaminergic system.

- Drug T is selective for H_2_ receptors, binds largely via hydrogen bonds, and mimics the effects of histamine.
- Drug W is selective for H_2_ receptors and binds via covalent bonds to block signal transduction.
- Drug Y is selective for H_2_ and H_3_ receptors, binds via hydrogen bonds, and blocks signal transduction.

Histamine acts on gastric parietal cells to stimulate gastric acid secretion. Critique the suitability of these three drugs for treating heartburn due to acid reflux and speculate about any unwanted effects they may produce.

**Kelsey** :- H_2_ receptors are located in the gastric mucosa, cardiac muscle, and mast cells. Thus, all three drugs will act on receptors located in these three locations, which may cause unwanted effects since we are only intending to target gastric parietal cells that stimulate gastric acid secretion. If Histamine acts on gastric parietal cells to stimulate gastric acid secretion, then to decrease the incidence of acid reflux in patients we would like to decrease the effects caused by histamine binding to H_2_ receptors in gastric mucosa.

Drug T would not be suitable to treat acid reflux since it mimics the effect of Histamine. That is, Drug T would similarly act on gastric parietal cells to stimulate gastric acid secretion. Drug T would increase gastric acid secretion, which is the opposite of what we want to happen to treat acid reflux.

Drug W would block the signal transduction of H_2_ receptors, thus decreasing the amount of gastric acid secretion, but it does so via covalent bonds which are not easily reversed. Thus, Drug W would cause a permanent block on H_2_ receptors. It is important to remember that H_2_ receptors are located not only in the gastric mucosa, but also in cardiac muscle and mast cells. Thus, it may be dangerous to permanently mask the effects of H_2_ receptors using Drug W since it would also affect cardiac muscle and mast cells, potentially causing unwanted and harmful side effects.

Lastly, Drug Y would decrease the amount of gastric acid secretion by blocking signal transduction in H_2_ receptors in the gastric mucosa. However, Drug Y, like Drug T and Drug W, will also affect H_2_ receptors in cardiac muscle and mast cells. In addition, Drug Y targets H_3_ receptors located in the CNS, myenteric plexus, and other neurons. Drug Y interacts with H_2_ and H_3_ receptors via hydrogen bonds, which makes its effect more transient as compared to covalent interactions (used by Drug W). However, there could still be unwanted or dangerous effects exhibited by Drug Y due to its interaction with H_3_ as well as H_2_ receptors.

***Question 4***

*A university student has given informed consent to participate in a pharmacokinetic study to determine the half-life of a novel anti-histamine in human subjects. Following drug administration, blood is drawn and serum concentrations (ng/ml) are determined as shown:*

- *10:00am 244*
- *11:00am 203*
- *12:00pm 171*
- *1:00pm 146*
- *2:00pm 122*
- *3:00pm 104*

*Calculate the drug’s half-life and how many hours are required for the student’s serum concentration to fall below 10 ng/ml.*

**Justin** :- From 10:00pam to 2:00pm, the plasma drug concentration drops in half from 244ng/ml to 122ng/ml. Therefore, the half-life of the antihistamine is 4 hours. Half-live (t1/2) is the length of time over which the active drug concentration in the blood decreases to one half its value. It will take 5 half-lives to reach a plasma drug concentration of 7.625ng/ml:

1 half-life: 122ng/ml

2 half-lives: 61ng/ml

3 half-lives: 30.5ng/ml

4 half-lives: 15.25ng/ml

5 half-lives: 7.625ng/ml

(5 half-lives)*(4 hours for each half-life) = 20 hours

It will take approximately 20 hours after the first measurement for the the student’s serum concentration to fall below 10 ng/ml.

**Faculty Preceptor** :- Good work, Justin.

***Question 5***

*Many drugs, like the histamine receptor antagonists, target G-protein coupled receptors and inhibit coupling to intracellular effector molecules. However some drugs, like dexamethasone, bind to intracellular glucocorticoid receptors that then associate with DNA response elements and subsequently alter gene transcription.*

*a. Speculate about the onset of action of drugs that target glucocorticoid receptors compared to drugs that target GPCRs.*

**Eva** :- Drugs that target glucocorticoid (GC) receptors would be able to diffuse through the plasma membrane to their intracellular drug targets. Through binding with their target intracellular GC receptor, the drug would be able to directly influence gene expression. In contract, drugs that target GPCRs use second-messenger systems such as cAMP to exert their pharmacological effects.

**Kelsey** :- I would speculate that the onset of action of drugs that target glucocorticoid receptors inside the cell would be longer than the onset of action of drugs that target GPCRs. GPCRs activate protein kinases that can immediately affect cell metabolism by going around and phosphorylating enzymes, which can have direct and rapid effects on the cell. However, the glucocorticoid receptor response may be slower since gene transcription takes some time. Thus, while drugs acting on glucocorticoid receptors may have long term metabolic effects by altering gene transcription, they may have a longer onset of action compared to drugs that target GPCRs.

**Sena** :- I would like to add to Kelsey's response - GPCR triggers a cascade of reactions that result in signal amplification. Nuclear Receptor ligands do not show amplification behavior, which is probably another reason why hydrophobic ligands, such as GCs, act more slowly than ligands that bind to cell membrane receptors.

*b. Based on your understanding of the ability of drugs to cross the cell membrane, how would you expect the pharmacokinetic properties to differ between a drug that targets a histamine receptor versus a drug that targets a nuclear binding site.*

**Eva** :- Like other GPCRs, histamine receptors are cell-surface receptors. Therefore, drugs that interact with this receptor must be water-soluble and may be found in extracellular spaces. Histamines and other drugs that target cell-surface receptors possess different properties than those that target nuclear receptors. Drugs that target nuclear receptors must be able to cross the plasma membrane in order to reach their target. Since only small, hydrophobic substances are able to cross the lipid bilayer, drugs that target nuclear receptors must possess these qualities. One example that we learned about is androgenic steroids, which diffuse easily through the lipid bilayer.

The pharmacokinetic properties we learned about are absorption, distribution, metabolism and excretion. Absorption of a lipid-soluble drug will different from a water-soluble drug in that it can diffuse through plasma membranes. Water-soluble drugs would need to enter through aquaporins or use transporters in order to enter circulation. A lipid-soluble drug that easily diffuses through membrane may be distributed more easily throughout the body. I am assuming that lipophilic drugs would distribute more widely through the body's tissues. They would be more likely to be found in all types of tissues, including adipose tissue, and thus can be described as having a larger bioavailability.

In terms of metabolism and excretion, lipid soluble drugs might be more difficult to eliminate from the body than water-soluble drugs which could be excreted in the urine. Anyone else want to weigh in on this?
