# Supplementary material for: An innovative pharmacology curriculum for medical students: promoting higher order cognition, learner-centered coaching, and constructive feedback through a social pedagogy framework
Source: BMC Med Educ. 2021 Feb 5;21:90. doi: 10.1186/s12909-021-02516-y (PMC7863331; doi:10.1186/s12909-021-02516-y)
Supplement: Supplementary file 3 — Additional file 3. Small group pharmacology wiki grading rubric. [file 12909_2021_2516_MOESM3_ESM.docx]

**Additional file 3**: Small group pharmacology wiki grading rubric.

|  | **PASS** | **FAIL** |
| --- | --- | --- |
| **TIMELINESS** | Assignments were completed on time. | Assignments were not fully completed or overdue. |
| **QUALITY OF ANSWER** | Evidence that learner responses related appropriately to the faulty-developed minimally competent answer. | Lacking evidence that learner responses related appropriately to the faculty-developed minimally competent answer. |
| **AUTHORSHIP, CITATIONS, RESPECT** | Evidence that responses were original (*i.e.*, learners contributed using their own words and perspectives), with information sources being credited when applicable, and learners were courteous and respectful to their peers. | Lacking evidence that responses were original (*i.e.*, learners contributed using their own words and perspectives), with information sources being credited when applicable, and learners were courteous and respectful to their peers. |
