# Supplementary material for: An innovative pharmacology curriculum for medical students: promoting higher order cognition, learner-centered coaching, and constructive feedback through a social pedagogy framework
Source: BMC Med Educ. 2021 Feb 5;21:90. doi: 10.1186/s12909-021-02516-y (PMC7863331; doi:10.1186/s12909-021-02516-y)
Supplement: Supplementary file 4 — Additional file 4. Representative example of a final, summative wiki assessment submission. [file 12909_2021_2516_MOESM4_ESM.docx]

**Additional file 4:** Representative example of a final, summative wiki assessment submission

*Individual Final Summative Wiki Instructions*:

- Pick one of the 10 OTC medications listed: *Mucinex D* (expectorant & cough suppressant), *Plan B* (emergency contraceptive), *Prilosec OTC* (treats frequent heartburn), *Nurofen* (targeted relief from pain), *Kool Comfort* (provides cooling pain relief), *Dramamine* (motion sickness relief), *Dream Water* (natural sleep and relaxation shot), *Sudafed PE* (pain reliever, fever reducer, and nasal decongestant), *Nicorette gum* (effective craving relief), and *5-Hour Energy* (liquid energy shot).
- Your assignment is to evaluate one claimed therapeutic benefit (described in the parentheses above) using your knowledge of pharmacokinetics and pharmacodynamics and the consumer version of the Drug/Supplement Facts provided in your own personal wiki.

**Final Summative Wiki for Over-The-Counter Medication: Dream Water**

Dream Water markets itself as a drug-free natural sleep and relaxation aid that contains a proprietary blend of SleepStat^TM^: 135 mg of gamma amino butyric acid (GABA), 5 mg of

Melatonin, and 10 mg of 5-hydroxytryptophan (5-HTP) (DreamWater, 2018). I will translate the consumer version of the drug/supplement facts with respect to the pharmacodynamics and pharmacokinetics of GABA, which here is claimed to reduce anxiety and promote relaxation.

**Pharmacodynamics**:

GABA (ɣ-aminobutyric acid) is a neurotransmitter widely distributed within the central nervous system, and represents the major element in neuronal inhibition (Julio- Pieper *et al*., 2013).

There are two main GABA receptors within the brain: GABA_A_ and GABA_B_. GABA_A_ is the most well-studied of the two. It is a ligand-gated chloride ion channel expressed on the post-synaptic side of the cleft, with orthosteric and allosteric binding sites. With GABA release from the pre-synaptic terminal, and binding to GABA_A_, the Cl^-^ channel is activated and Cl^-^ enters the neuron, effectively hyperpolarizing the membrane and increasing the threshold for action potential generation.

Unlike GABA_A_, GABA_B_ is a G-protein-coupled receptor. GABA signaling here decreases calcium conductance, inhibits cAMP production, increases K^+^ efflux – the net effect of which will hyperpolarize and reduce the frequency of post-synaptic action potentials (Kumar *et al*, 2015. *Neuropharmacology*. 97:414-425). Thus, by triggering the inhibitory effects of GABAergic neurons in the central nervous system, the GABA added to Dream Water could potentially exert anxiolytic and myorelaxant effects (Richard and Foster, 2003; Chou *et al*., 2008; Mazzoli *et al*., 2010).

**Pharmacokinetics**:

GABA is a neurotransmitter generated within the body by the enzyme GAD (glutamic acid decarboxylase) from Glu (Mazzoli and Pessione, 2016). After exerting its function within the synaptic cleft, GABA experiences reuptake, whereby the presynaptic terminal and surrounding supporting glia cells reabsorb it in an exchange with sodium. The GABA which is reabsorbed by the neuron can be reused, while the GABA reabsorbed by the glia is metabolized to succinic semialdehyde and must be regenerated by GAD. (Cherlyn *et al*., 2010; Femenía *et al*., 2012) Under non-pathologic conditions, the body is able to synthesize and recycle the entirety of its GABA needs.

Exogenously administered GABA, as in the case of DreamWater, would be subject to the challenges of absorption, distribution, metabolism, and excretion, as any exogenous substance is. GABA’s capacity to traverse the blood-brain barrier (BBB) is controversial (Boonstra et al. 2015). The bulk of evidence supports the impermeability of BBB to GABA, although Boonstra et al. (2015) report that “BBB permeability to GABA could diminish with increasing age.” In addition, mouse model studies have demonstrated evidence of GABA-facilitated transport across the BBB; however, measurements of GABA efflux were 17x greater than GABA-facilitated influx (Kakee et al. 2001). The presence of equivalent GABA transporters in the human blood-brain barrier has yet to be established. Moreover, Knych *et al* (2015) demonstrated in a horse animal model that exogenous oral GABA – with a half-life of 25 minutes, bioavailability of 9.8%, and urine concentration peaking at 3 hours – had no discernable behavior effect. This means that half of the given GABA had been metabolized within 25 minutes, and that of all of the GABA administered, only 9.8% was available as active within the body. Given that no behavioral changes where seen with exogenous oral GABA, but was with IV, it appears that it did not appreciably enter the cerebrospinal fluid to exert its effects. Therefore, the ultimate benefit of the GABA added to DreamWater may be in question. A Phase I clinical trial to study exogenous GABA in humans is currently recruiting participants (https://clinicaltrials.gov/ct2/show/NCT01917760).

**References**:

DreamWater (2018) Available at https://usa.drinkdreamwater.com/pages/how-it-works; accessed Mar 16, 2018).

Kumar M, González LA, Dillon GH (2015). Assessment of subunit-dependent direct gating and allosteric modulatory effects of carisoprodol at GABAA receptors. *Neuropharmacology*. 97:414-425

Mazzoli R and Pessione E (2016). The Neuro-endocrinological Role of Microbial Glutamate and GABA Signaling. *Frontiers in Microbiology.* Vol 7: Article 1934

Julio-Pieper M, O’Connor RM, Dinan TG, and Cryan JF (2013). Regulation of the brain-gut axis by group III metabotropic glutamate receptors. *Eur. J. Pharmacol.* 698, 19–30.

Soeiro-de-Souza MG, Henning A, Machado-Vieira R, Moreno RA, Pastorello B F, da Costa, *et al*. (2015). Anterior cingulate glutamate- glutamine cycle metabolites are altered in euthymic bipolar I disorder. *Eur. Neuropsychopharmacol.* 25, 2221–2229.

Roth FC and Draguhn A (2012). GABA metabolism and transport: effects on synaptic efficacy. *Neural Plast.* 2012:805830.

Richard HT and Foster JW (2003). Acid resistance in *Escherichia coli*. *Adv. Appl. Microbiol.* 52, 167–186.

Mazzoli R, Pessione E, Dufour M, Laroute V, Giuffrida MG, Giunta C, *et al*. (2010). Glutamate-induced metabolic changes in *Lactococcus lactis* NCDO 2118 during GABA production: combined transcriptomic and proteomic analysis. *Amino Acids* 39, 727–737.

Chou HT, Kwon DH, Hegazy M and Lu CD (2008). Transcriptome analysis of agmatine and putrescine catabolism in *Pseudomonas aeruginosa* PAO1. *J. Bacteriol.* 190, 1966–1975.

Lee AC, Godfrey DA. Cochlear damage affects neurotransmitter chemistry in the central auditory system. *Front Neurol*. 2014;5:227

Cherlyn SY, Woon PS, Liu JJ, Ong WY, Tsai GC, and Sim K (2010). Genetic association studies of glutamate, GABA and related genes in schizophrenia and bipolar disorder: a decade of advance. *Neurosci. Biobehav. Rev.* 34, 958–977.

Femenía T, Gómez-Galán M, Lindskog M, and Magara S (2012). Dysfunctional hippocampal activity affects emotion and cognition in mood disorders. *Brain Res.* 1476, 58–70.

Knych HK, Steinmetz SJ, Mckemie DS. Endogenous concentrations, pharmacokinetics, and selected pharmacodynamic effects of a single dose of exogenous GABA in horses. *J Vet Pharmacol Ther*. 2015;38(2):113-22

Boonstra E, de Kleijn R, Colzato LS, Alkemade A, Forstmann BU, and Nieuwenhuis S (2015). Neurotransmitters as food supplements: the effects of GABA on brain and behavior. *Front. Psychol.* 6:1520.

Kakee A, Takanaga H, Terasaki T, Naito M, Tsuruo T, and Sugiyama Y (2001). Efflux of a suppressive neurotransmitter, GABA, across the blood-brain barrier. *J. Neurochem.* 79, 110–118.
