# Supplementary material for: An innovative pharmacology curriculum for medical students: promoting higher order cognition, learner-centered coaching, and constructive feedback through a social pedagogy framework
Source: BMC Med Educ. 2021 Feb 5;21:90. doi: 10.1186/s12909-021-02516-y (PMC7863331; doi:10.1186/s12909-021-02516-y)
Supplement: Supplementary file 5 — Additional file 5. Summative Pharmacology MCQ Item Statistics. [file 12909_2021_2516_MOESM5_ESM.docx]

**Additional file 5:** Summative Pharmacology MCQ Item Statistics

Key:

P-value = percentage of learners that answered correctly

Pbis = point biserial correlation

FOS I = Foundations of Science I

FOS II = Foundations of Science II

MSK&I = Musculoskeletal & Integument

NH&N = Neuroscience, Head & Neck Anatomy

HLK = Heart, Lung & Kidney

GIGUR = Gastrointestinal, Genitourinary & Reproductive
