# Supplementary material for: An innovative pharmacology curriculum for medical students: promoting higher order cognition, learner-centered coaching, and constructive feedback through a social pedagogy framework
Source: BMC Med Educ. 2021 Feb 5;21:90. doi: 10.1186/s12909-021-02516-y (PMC7863331; doi:10.1186/s12909-021-02516-y)
Supplement: Supplementary file 6 — Additional file 6. Percentage of students who failed formative small group wiki assignments and the percentage of failures due to timeliness, quality or authorship/citations/respect for M1 students 2014–2018. [file 12909_2021_2516_MOESM6_ESM.docx]

**Additional file 6:** Percentage of students who failed formative small group wiki assignments and the percentage of failures due to timeliness, quality or authorship/citations/respect for M1 students 2014-2018.
